# Characterization of a Gene Encoding Clathrin Heavy Chain in Maize Up-Regulated by Salicylic Acid, Abscisic Acid and High Boron Supply

**DOI:** 10.3390/ijms140715179

**Published:** 2013-07-22

**Authors:** Mu-Heng Zeng, Sheng-Hong Liu, Miao-Xian Yang, Ya-Jun Zhang, Jia-Yong Liang, Xiao-Rong Wan, Hong Liang

**Affiliations:** College of Life Sciences, Zhongkai University of Agriculture and Engineering, Guangzhou 510225, China; E-Mails: mhzengzk@126.com (M.-H.Z.); sh-liu01@163.com (S.-H.L.); amiao_731@sina.com (M.-X.Y.); yajunzhang2005@163.com (Y.-J.Z.); ljysky2004@sina.com (J.-Y.L.)

**Keywords:** clathrin heavy chain, gene cloning and expression, salicylic acid and abiotic stresses, maize (*Zea mays* L.)

## Abstract

Clathrin, a three-legged triskelion composed of three clathrin heavy chains (CHCs) and three light chains (CLCs), plays a critical role in clathrin-mediated endocytosis (CME) in eukaryotic cells. In this study, the genes *ZmCHC1* and *ZmCHC2* encoding clathrin heavy chain in maize were cloned and characterized for the first time in monocots. *ZmCHC1* encodes a 1693-amino acid-protein including 29 exons and 28 introns, and *ZmCHC2* encodes a 1746-amino acid-protein including 28 exons and 27 introns. The high similarities of gene structure, protein sequences and 3D models among *ZmCHC1*, and Arabidopsis *AtCHC1* and *AtCHC2* suggest their similar functions in CME. *ZmCHC1* gene is predominantly expressed in maize roots instead of ubiquitous expression of *ZmCHC2*. Consistent with a typical predicted salicylic acid (SA)-responsive element and four predicted ABA-responsive elements (ABREs) in the promoter sequence of *ZmCHC1*, the expression of *ZmCHC1* instead of *ZmCHC2* in maize roots is significantly up-regulated by SA or ABA, suggesting that *ZmCHC1* gene may be involved in the SA signaling pathway in maize defense responses. The expressions of *ZmCHC1* and *ZmCHC2* genes in maize are down-regulated by azide or cold treatment, further revealing the energy requirement of CME and suggesting that CME in plants is sensitive to low temperatures.

## 1. Introduction

In eukaryotic cells, endocytosis is essential for regulation of the protein and lipid compositions of the plasma membrane and for acquisition or removal of material from the extracellular medium [1,2]. In animal cells, depending on the machinery involved, endocytosis is roughly classified into clathrin-mediated (CME) and clathrin-independent (CIE) endocytosis [1,3–5]. CME initiates at the plasma membrane with the recruitment of cargo and the coat machinery into foci called clathrin coated pits (CCPs) that eventually mature and scission off to form clathrin coated vesicles (CCVs). The CCVs are uncoated in seconds to form uncoated vesicles that fuse with the early endosome (EE) where the cargo is further sorted, either for recycling back to the plasma membrane, or kept in the endocytic pathway to later endosomes, lysosomes and vacuoles for degradation [4,5]. Similar internalizing mechanisms exist in plant cells [2], however, compared with the elegant CME network in animals, the core composition and regulation of the endocytic machinery in plants is still poorly defined [5,6].

Clathrin is a protein that plays a major role in the formation of coated vesicles. Clathrin was first isolated and named by Barbara Pearse in 1976 [7]. It forms a hexameric, three legged triskelion composed of three clathrin heavy chains (CHCs) and three light chains (CLCs) [8]. The three clathrin heavy chains, interacting at their *C*-termini, provide the structural backbone of the clathrin lattice, and the three light chains are thought to regulate the formation and disassembly of a clathrin lattice. Clathrin heavy chain is, in concept, broken down into multiple subdomains, starting with the *N*-terminal domain, followed by the ankle, distal leg, knee, proximal leg, and trimerization domains. The *N*-terminal domain consists of a seven-bladed β-propeller structure. The other domains form a super-helix of short alpha helices. The light chains bind primarily to the proximal leg portion of the heavy chain with some interaction near the trimerization domain [5,6,8].

Endocytosis in plants has an essential role not only for basic cellular functions but also for growth and development, hormonal signaling and communication with the environment including nutrient delivery, toxin avoidance, and pathogen defense [5]. The major endocytic mechanism in plants depends on the coat protein clathrin. It starts by clathrin-coated pit (CCP) and vesicle (CCV) formation at the plasma membrane, where specific cargoes are recognized and packaged for internalization [5]. Recently, the role of CME was molecularly demonstrated as a major route of endocytosis in plant cells [6,9–11]. So far, the gene encoding clathrin heavy chain in higher plant cells was only characterized in soybean [12] and Arabidopsis [11], and the gene encoding clathrin light chain was solely characterized in Arabidopsis [5,11]. Arabidopsis encodes two *CHC* and three *CLC* genes [11]. Plasma membrane localization of clathrin and genetic analysis of *chc* loss of function mutants and dominant negative clathrin lines in Arabidopsis [9–11,13] firmly established the conserved mechanism of CME as a fundamental process required for plant growth and development. However, the physiological and developmental importance of clathrin-mediated endocytosis in plants, particularly in monocots, awaits detailed characterization.

Maize is one of the world’s most important food sources with a total world production of ~827 × 10^6^ metric tons [14]. It ranks with rice (689 × 10^6^ tons) and wheat (683 × 10^6^ tons) among the world’s most cultivated crop plants. Worldwide, significant efforts are underway to use modern biotechnology to assist maize breeding programs to increase crop yield, nutritional content, salinity and drought tolerance, as well as biotic tolerance [15]. In the present study, we report the cloning and characterization of two genes encoding clathrin heavy chain in monocot plant maize, designated as *ZmCHC1* (GenBank accession no. KC006065) and *ZmCHC2* (GenBank accession no. BK008734), respectively. The *ZmCHC1* and *ZmCHC2* genes are respectively composed of 16,926 bp encoding a 1693-amino acid-protein including 29 exons and 28 introns, and 12,370 bp encoding a 1746-amino acid-protein including 28 exons and 27 introns. Real-time RT-PCR performance shows the organ specific expression pattern of *ZmCHC1* and the ubiquitous expression of *ZmCHC2*. The expression of *ZmCHC1* gene in maize roots is significantly up-regulated by salicylic acid (SA), abscisic acid (ABA) or high boron levels, suggesting that *ZmCHC1* gene may be involved in the SA signaling pathway in maize defense responses. The expressions of *ZmCHC1* and *ZmCHC2* genes in maize are significantly down-regulated by azide or cold treatment, further revealing the energy requirement of CME and suggesting that CME in plants is sensitive to low temperatures.

## 2. Results

### 2.1. Cloning and Characterization of Genes *ZmCHC1* and *ZmCHC2* Encoding Clathrin Heavy Chain in *Zea Mays*

**Figure 1:**
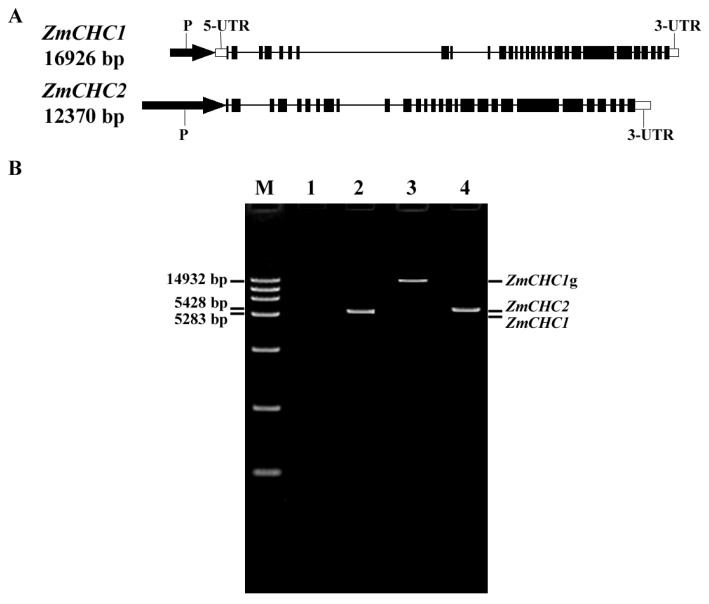
(**A**) Schematic representation of the intron (bar)-exon (black box) structure in maize *ZmCHC1* and *ZmCHC2* genes. P denotes the promoter region; UTR is the untranslated region; (**B**) RT-PCR performance amplifying the sequence containing complete ORF of *ZmCHC1* (Lane 2) or *ZmCHC2* (Lane 4) with maize root cDNA or water (Lane 1) as the template. Lane 3 (*ZmCHC1*g) was a PCR product of corresponding genomic DNA of *ZmCHC1*. M: DL 15000 DNA Marker (TaKaRa).

The conserved regions of the reported clathrin heavy chain proteins (CHCs) were used for the design of degenerate primers used in the polymerase chain reaction (PCR) amplification of new *CHC* homolog from maize plants. Two fragments (named as *ZmCHC1* and *ZmCHC2*, respectively) were amplified from the cDNA of maize roots. The sequences of the two fragments show high similarity with the reported *CHC*s in GenBank DNA database. The full length cDNA of *ZmCHC1* obtained through RACE, consists of 5765 bp nucleotides, including a 372-bp 5′ untranslated region (5′ UTR) and a 311-bp 3′ untranslated region (3′ UTR). *ZmCHC1* cDNA has an open reading frame (ORF), encoding a polypeptide of 1693 amino acid residues with a calculated molecular weight of 192.08 kDa and an isoelectric point of 5.21. The genomic DNA sequence corresponding to the coding sequence of *ZmCHC1* was amplified using a LA Taq DNA polymerase (TaKaRa), and a 1512-bp promoter region sequence of *ZmCHC1* was cloned by using the Genome Walking Kit (TaKaRa). The *ZmCHC1* gene is composed of 16,926 bp including a 1512-bp promoter sequence, a 372-bp 5′ UTR, a complete open reading frame encoding a 1693-amino acid-protein with 29 exons and 28 introns, and a 311-bp 3′ UTR (Figure 1A). The full length cDNA of *ZmCHC2* (GenBank accession no. KC691697) obtained through RACE, consists of 5616 bp nucleotides, including a 375-bp 3′ UTR. *ZmCHC2* cDNA has an open reading frame, encoding a polypeptide of 1746 amino acid residues with a calculated molecular weight of 197.93 kDa and an isoelectric point of 5.29. The corresponding genomic DNA sequence of *ZmCHC2* was achieved by comparison of *ZmCHC2* cDNA sequence with the submitted maize chromosome sequence (GenBank accession no. CM000778). The *ZmCHC2* gene is composed of 12,370 bp including a 2048-bp promoter sequence (2048 bp upstream of the start codon of *ZmCHC2*), a complete open reading frame encoding a 1746-amino acid-protein with 28 exons and 27 introns, and a 375-bp 3′ UTR (Figure 1A). RT-PCR was performed to detect the possible transcript variants of *ZmCHC1* and *ZmCHC2* genes. All the nested RT-PCR products using the primers CHC1F-inner and CHC1R-inner were subcloned and sequenced to be one *ZmCHC1* transcript of 5283 bp (Figure 1B). Compared with the corresponding 14,932-bp genomic DNA sequence of *ZmCHC1*, alternative splicing should not exist in the coding sequence of *ZmCHC1* gene during transcription (Figure 1B). For *ZmCHC2* gene, all the nested RT-PCR products using the primers CHC2F-inner and CHC2R-inner were subcloned and sequenced to be one *ZmCHC2* transcript of 5428 bp (Figure 1B). Compared with the corresponding maize chromosomal DNA sequence (GenBank accession no. CM000778), alternative splicing should also not exist in the coding sequence of *ZmCHC2* gene during transcription. The complete sequences of *ZmCHC1* and *ZmCHC2* genes have been deposited in the GenBank nucleotide sequence database under the accession numbers KC006065 and BK008734, respectively.

The promoter region sequences of *ZmCHC1* (1500 bp upstream of the start codon of *ZmCHC1*) and *ZmCHC2* (1500 bp upstream of the start codon of *ZmCHC2*) genes were submitted to PlantCARE, a database of plant *cis*-acting regulatory elements [16], to predict the *cis*-acting elements that may regulate the expressions of *ZmCHC1* and *ZmCHC2* in maize. The results show that there are many important *cis*-acting elements, including TATA-box, CAAT-box, ABA-responsive element (ABRE), fungal elicitor responsive element, and methyl jasmonate (Me-JA) responsive element in both of the promoter sequences. Furthermore, a typical salicylic acid (SA)-responsive element (TCA-element) was predicted in the promoter sequence of *ZmCHC1* instead of *ZmCHC2* (Table 1). The prediction of *cis*-acting elements in promoter region sequences of *ZmCHC1* and *ZmCHC2* genes provides indicating information about the regulation of the two genes’ expression in maize plants. The main *cis*-acting elements of the prediction results were listed in Table 1.

### 2.2. Sequence Analysis of ZmCHC1 and ZmCHC2

**Figure 2:**
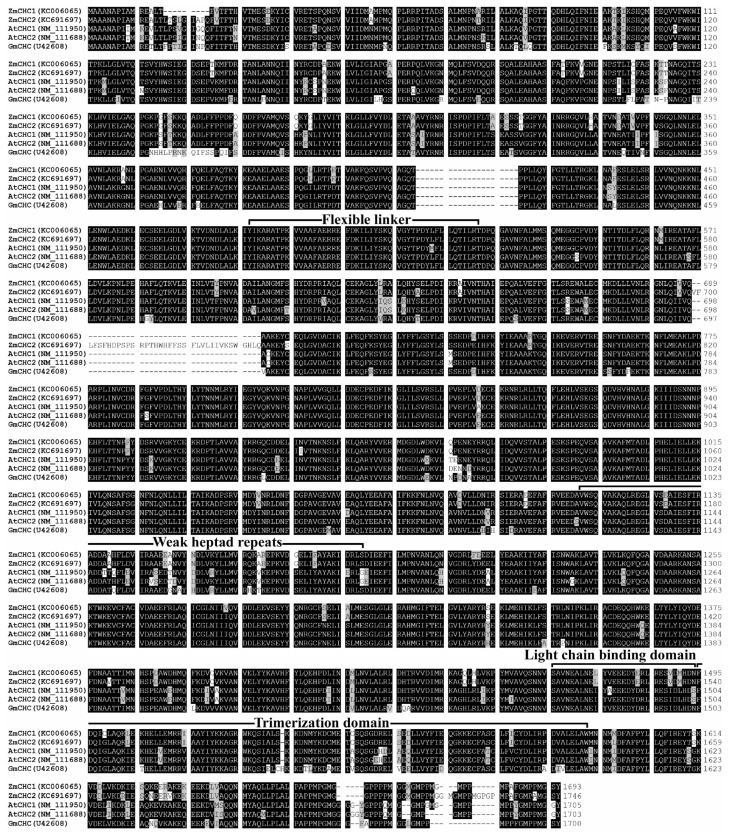
Alignment of deduced amino acid sequences of maize ZmCHC1, ZmCHC2 with Arabidopsis AtCHC1, AtCHC2 and soybean GmCHC. Identical and similar amino acid residues were shaded in black and gray, respectively. The reported consensus domains including flexible linker, weak heptad repeats, light chain binding and trimerization, were indicated. GenBank accession numbers for each aligned CHC were indicated in parentheses. Amino acids were numbered from the initial methionine.

**Figure 3:**
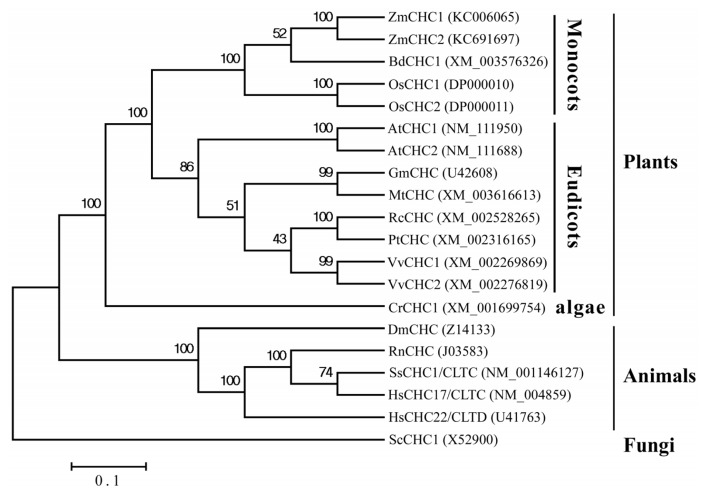
Phylogenetic analysis of the amino acid sequences of maize ZmCHC1, ZmCHC2 and other eukaryotic CHC proteins deposited in the Genbank database. Multiple sequence alignment was performed using Clustal W and the phylogenetic tree was constructed via the Neighbor-Joining method in MEGA 4 software. Bootstrap values from 1000 replicates for each branch were shown. GenBank accession numbers for each analyzed CHC were indicated in parentheses. The scale bar is 0.1. At, *Arabidopsis thaliana*; Bd, *Brachypodium distachyon*; Cr, *Chlamydomonas reinhardtii*; Dm, *Drosophila melanogaster*; Gm, *Glycine max*; Hs, *Homo sapiens*; Mt, *Medicago truncatula*; Os, *Oryza sativa*; Pt, *Populus trichocarpa*; Rc, *Ricinus communis*; Rn, *Rattus norvegicus*; Sc, *Saccharomyces cerevisiae*; Ss, *Sus scrofa*; Vv, *Vitis vinifera*.

Up until now, the genes encoding clathrin heavy chain have only been well characterized in rat [17], yeast [18], human [19,20], *Drosophila* [21,22], soybean [12], pig [23], and Arabidopsis [11]. Multiple sequence alignments of the deduced amino acids of ZmCHC1 and ZmCHC2 showed that ZmCHC1 and ZmCHC2 shared 96.1% sequence identity with each other, and that ZmCHC1 shared 90.3%, 89.9%, and 88.9% sequence identity with Arabidopsis AtCHC1, AtCHC2, and soybean GmCHC, respectively. There is evidence that the light chain binding and trimerization domains of mammalian CHCs lie adjacent to each other, spanning residues 1460–1489 and 1490–1587, respectively [24]. Multiple sequence alignments revealed that the light chain binding and trimerization domains were well conserved in maize ZmCHC1 and ZmCHC2 (Figure 2). The light chain binding domain spanned residues 1466–1494 in ZmCHC1 or residues 1511–1539 in ZmCHC2. The trimerization domain aligned to the residues 1495–1592 in ZmCHC1 or residues 1512–1609 in ZmCHC2. The *N*- and *C*-terminal domains, flexible linker (residues 483–528 in ZmCHC1 or residues 492–537 in ZmCHC2), and weak heptad repeats (residues 1112–1189 in ZmCHC1 or residues 1157–1234 in ZmCHC2) like in soybean GmCHC [12], were also found in both ZmCHC1 and ZmCHC2 (Figure 2). Phylogenetic analysis of ZmCHC1, ZmCHC2 and other eukaryotic CHC proteins deposited in the Genbank database were performed by using the Neighbor-Joining method in MEGA 4 software [25]. The results showed that the eukaryotic CHC proteins can be grouped into fungi, plant and animal clusters (Figure 3). *ZmCHC1* and *ZmCHC2* were phylogenetically classified into the monocots group in the plants cluster (Figure 3).

**Figure 4:**
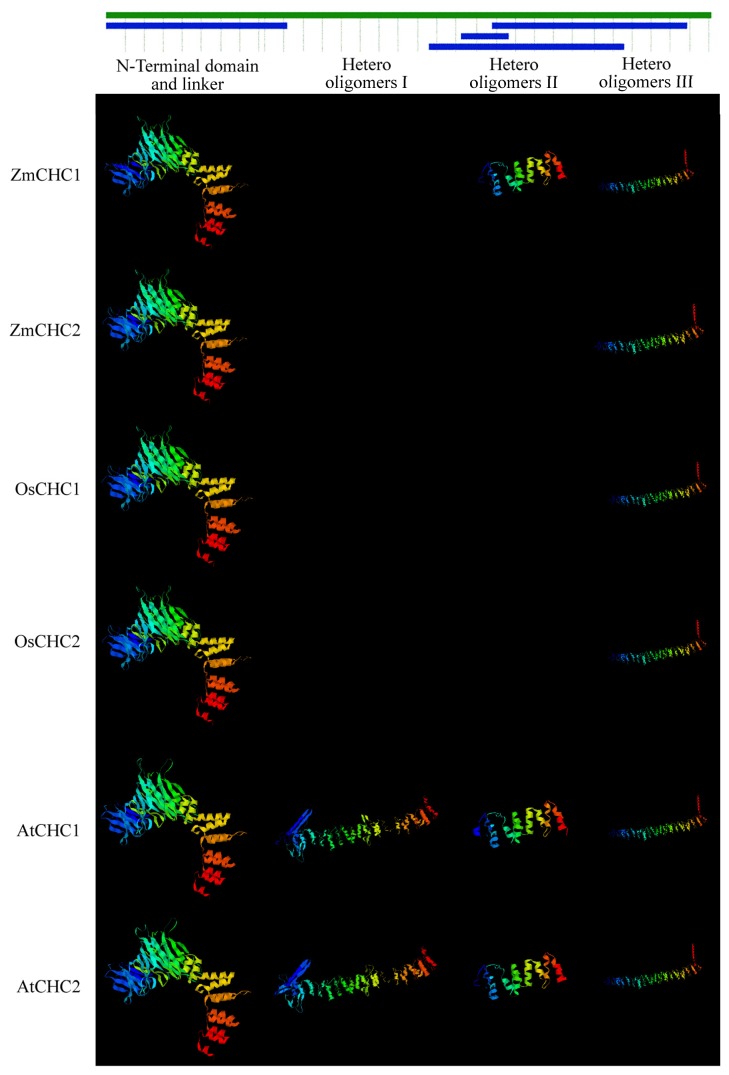
Ribbon diagrams of major 3D structures of plant CHC proteins generated with SWISS-MODEL. Green bars above the ribbon diagrams represent protein sequences with their corresponding amino acid lengths, and blue bars indicate the sequence areas where 3D structures were generated with SWISS-MODEL. Structures were color-coded ranging from the *N*-terminus (blue) to the *C*-terminus (red). ZmCHC1 and ZmCHC2 from maize, OsCHC1 and OsCHC2 from rice, AtCHC1 and AtCHC2 from Arabidopsis were used in the 3D models analysis. The SWISS-MODEL tool did not generate a 3D structure for hetero oligomers I and II in ZmCHC2, OsCHC1 and OsCHC2, and did not generate a 3D structure for hetero oligomers I in ZmCHC1.

Comparison of 3D models with SWISS-MODEL Workspace revealed that similar 3D structures existed among maize ZmCHC1 and ZmCHC2, rice OsCHC1 and OsCHC2, and Arabidopsis AtCHC1 and AtCHC2 (Figure 4). 3D structure analysis indicated that the *N*-terminal domain and linker, and hetero oligomers III were generated in all CHC proteins analyzed. The 3D structures for *N*-terminal domain and linker, and hetero oligomers II and III were all generated in ZmCHC1, and Arabidopsis AtCHC1 and AtCHC2. The SWISS-MODEL tool did not generate a 3D structure for hetero oligomers I in all monocots CHCs analyzed, including ZmCHC1, ZmCHC2, OsCHC1 and OsCHC2, implying that the hetero oligomers I might be a unique 3D structure of CHCs in eudicots. The 3D structures for hetero oligomers I and II were not generated in ZmCHC2, and rice OsCHC1 and OsCHC2 (Figure 4). ZmCHC1 is predicted as not having any of signal, mitochondrial targeting, or chloroplast transit peptides by the iPSORT prediction [26], and that ZmCHC2, AtCHC2 and GmCHC are predicted as having a mitochondrial targeting peptide (MAAANAPIAMREALTLTSLGIAPQFVTFTH, MAAANA PITMKEVLTLPSIGINQQFITFTN and MAAANAPIAMRETLTFPTIGINPQFITFTH for ZmCHC2, AtCHC2 and GmCHC, respectively). AtCHC1 is predicted as having a chloroplast transit peptide (MAAANAPIIMKEVLTLPSVGIGQQFITFTN). In our subsequently ongoing study, the binary vector harboring the sequence encoding N terminal 60 amino acids of ZmCHC1 or ZmCHC2 fusioned with *GFP* from pCAMBIA1302 was constructed, and the analysis of transient expression of GFP will tell us the exact subcellular localization of ZmCHC1 and ZmCHC2 proteins.

### 2.3. Organ Specific Expression Patterns of *ZmCHC1* and *ZmCHC2* in Maize Seedling

**Figure 5:**
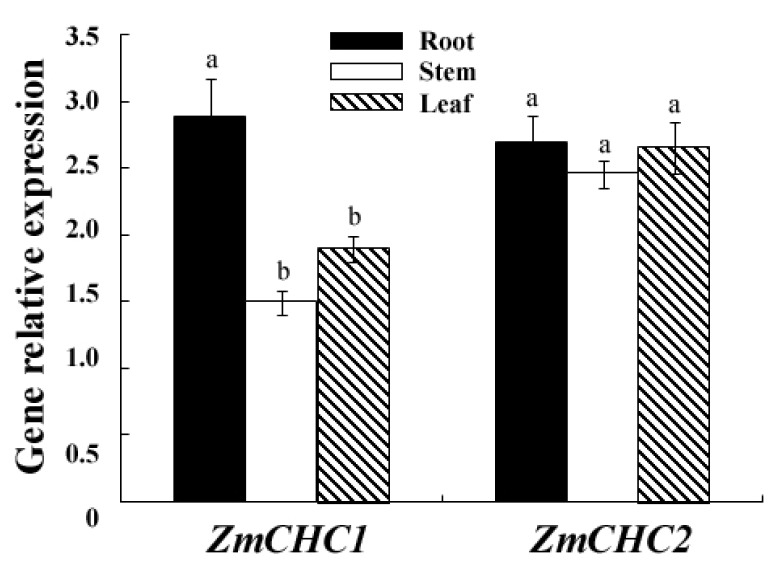
Expressions of *ZmCHC1* and *ZmCHC2* genes in maize plants under normal conditions were determined by real-time RT-PCR and were shown relative to the expression of maize *18S rRNA* gene in each sample. Total RNA was prepared respectively from root, stem, and leaf of maize plants. All data are presented as mean ± standard errors (SE) of three replicates. A different letter above each bar indicates a significant difference between organs (*p* < 0.05, one-way ANOVA and LSD/SNK *post hoc* test).

Real-time quantitative RT-PCR analysis was performed to examine the expressions of *ZmCHC1* and *ZmCHC2* in 12-day-old maize seedlings (Figure 5). The results showed that *ZmCHC1* and *ZmCHC2* were expressed in all organs examined, including roots, stems and leaves. However, the *ZmCHC1* gene transcript level in roots of maize was relatively higher than that in leaves and stems. The *ZmCHC2* gene was ubiquitously expressed in leaves, stems and roots of maize. There is no significant difference in the transcript abundance between *ZmCHC1* and *ZmCHC2* in maize roots.

### 2.4. Effect of ABA, High Boron Supply, Sodium Azide or Low Temperature on Expressions of *ZmCHC1* and *ZmCHC2* in Maize Plants

**Figure 6:**
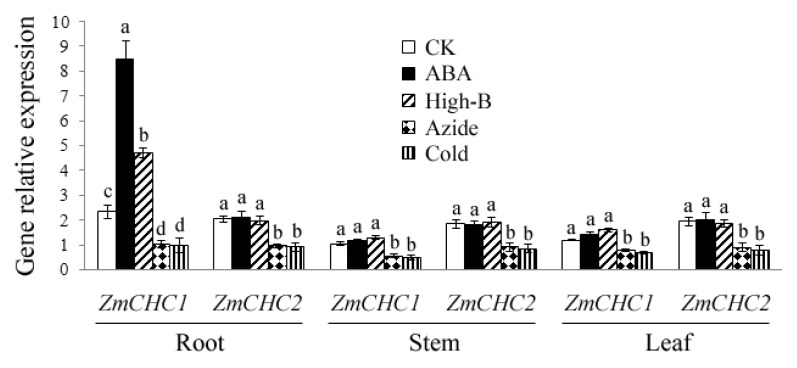
Organ specific expressions of *ZmCHC1* and *ZmCHC2* in maize seedling in response to exogenous abscisic acid (ABA), high boron supply (High-B), sodium azide, or low temperature (Cold) were determined by real-time quantitative RT-PCR performance, and were shown relative to the expression of maize *18S rRNA* gene in each sample. Total RNA was prepared respectively from root, stem and leaf of treated or control (CK) maize plants. All data are presented as mean ± SE of three replicates. A different letter above each bar indicates a significant difference between treatments (*p* < 0.05, one-way ANOVA and LSD/SNK *post hoc* test).

The expressions of *ZmCHC1* and *ZmCHC2* genes in maize plants in response to exogenous abscisic acid (ABA), high boron levels, sodium azide, or low temperature were determined by real-time quantitative RT-PCR performance. As shown in Figure 6, the expression of *ZmCHC1* gene in roots was significantly up-regulated by exogenous ABA or high boron supply, whereas the transcript levels of *ZmCHC1* in leaves and stems only slightly increased by those treatments. The transcript level of *ZmCHC2* gene in maize plants was not influenced by exogenous ABA or high boron supply. The expressions of *ZmCHC1* and *ZmCHC2* genes in maize seedlings were both significantly down-regulated by sodium azide or low temperature (Figure 6).

### 2.5. Salicylic Acid-Inducible Expression of *ZmCHC1* Gene in Maize Roots

**Figure 7:**
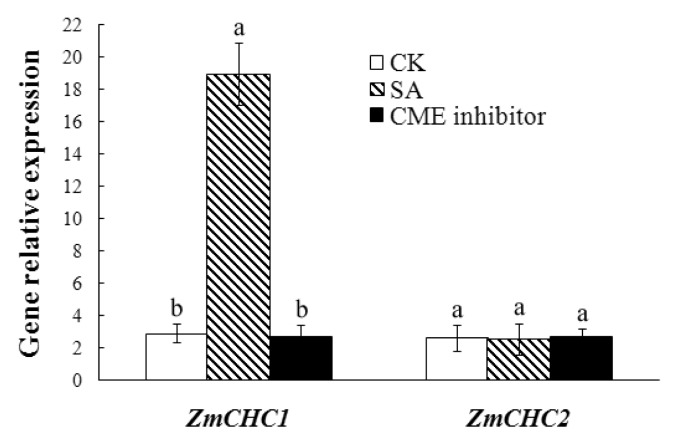
Expressions of *ZmCHC1* and *ZmCHC2* genes in maize roots in response to salicylic acid (SA) and Tyrphostin A23, a commonly used inhibitor of clathrin-mediated endocytosis, were determined by real-time quantitative RT-PCR, and were shown relative to the expression of maize *18S rRNA* gene in each sample. Total RNA was prepared from roots of treated or control (CK) maize plants. All data are presented as mean ± SE of three replicates. A different letter above each bar indicates a significant difference between treatments (*p* < 0.01, one-way ANOVA and LSD/SNK *post hoc* test).

Salicylic acid (SA) plays an important regulatory role in multiple physiological processes such as the plant immune response. Furthermore, SA can interact with other phytohormones such as ABA, auxin, and gibberellic acid [27,28]. It has been recognized that SA is required in the signal transduction for inducing systemic acquired resistance against some pathogenic infections [29]. Real-time quantitative RT-PCR analysis was performed using total RNA from roots of 12-day-old maize seedlings exposed to 100 μmol/L SA or 5 μmol/L Tyrphostin A23, a commonly used inhibitor of clathrin-mediated endocytosis (CME) [5,10], to examine whether the expressions of *ZmCHC1* and *ZmCHC2* genes were regulated by SA or CME inhibitors (Figure 7). The results showed that the transcript level of *ZmCHC1* gene increased by 6.58-fold in maize roots in response to SA treatment compared with that in the control roots. The expression level of *ZmCHC1* gene was not affected by the CME inhibitor Tyrphostin A23. No obvious changes were observed in the expression of *ZmCHC2* gene after either of the SA and Tyrphostin A23 treatments (Figure 7).

## 3. Discussion

Clathrin-mediated endocytosis is the endocytic portal into cells through which cargo is packaged into vesicles with the aid of the clathrin coat. It is fundamental to signal transduction and the regulation of many plasma membrane activities and is thus essential to higher eukaryotic life. Similar cargo sorting mechanisms exist in plant cells, but the mechanisms involved in internalization have been unclear and need further investigation [2,30]. Dhonukshe *et al*. [9] showed that clathrin is required for internalization of a wide variety of plasma membrane markers, implicating clathrin-coated vesicles as a major internalization mechanism in plants. Pharmacological and genetic data indicate that endocytic recycling is important for maintenance and dynamic modulation of cell polarity with respect to auxin transporters at the plasma membrane [31], and may also play a role in signaling or modulation of cellular sensitivity, as in animal cells [1,32]. In the present study, two genes, *ZmCHC1* and *ZmCHC2*, encoding clathrin heavy chain were cloned and characterized from maize plants. The *ZmCHC1* and *ZmCHC2* genomic DNA sequences respectively contain a 1512-bp promoter sequence, an ORF encoding a 1693-amino acid-protein with 29 exons and 28 introns, and a 2048-bp promoter region sequence, an ORF encoding a 1746-amino acid-protein with 28 exons and 27 introns (Figure 1). The similar gene structure organization was recently reported in Arabidopsis *AtCHC1* (containing 30 exons and 29 introns) and *AtCHC2* (containing 24 exons and 23 introns) [11]. The prediction of *cis*-acting regulatory elements through PlantCARE shows that four possible ABREs exist in the promoter sequence of *ZmCHC1* gene, and that only one exists in *ZmCHC2* gene. A fungal elicitor responsive element (Box-W1) was predicted in both of the two promoter sequences. The typical salicylic acid (SA)-responsive element (TCA-element) was predicted in the promoter sequence of *ZmCHC1* instead of *ZmCHC2* gene (Table 1). The different *cis*-acting elements in promoter region sequences of *ZmCHC1* and *ZmCHC2* genes may explain the difference in the expression of *ZmCHC1* and *ZmCHC2* in response to SA and ABA (Figures 6 and 7).

The *C*-terminal domain of CHCs (residues 1590–1675 in mammals) may form the globular protrusion on top of triskelia at the vertex [24]. It is evident that the residues at the extreme *C*-termini are very poorly conserved between species. Analysis of a *C*-terminal mutant of yeast HC has already demonstrated that this globular protrusion plays no essential role in triskelia assembly or clathrin function [18]. An analysis of clathrin heavy chain and light chain sequences suggests that there is a high probability of their interacting via α-helical coiled-coils [24]. Multiple sequence alignments show that both of the two maize CHCs share high similarity with previously reported CHCs in database (Figure 2). The high degree of sequence identity (96.1%) between ZmCHC1 and ZmCHC2 hints at functional redundancy between both of the maize *CHC* genes (Figure 2), as recently reported in Arabidopsis *AtCHC1* and *AtCHC2* genes [11]. The flexible linker, light chain binding and trimerization domains of ZmCHC1 and ZmCHC2 are highly conserved to those domains in CHCs characterized in only two other plants (Figure 2), soybean [12] and Arabidopsis [11]. The flexible linker (residues 479–523 in rat CHC), also found in both of ZmCHC1 and ZmCHC2 (Figure 2), allows the globular *N*-terminal domain to project inwards and make contact with the adaptor complex [17]. The weakly predicted pattern of heptad repeats spanning residues 1107–1184 in rat CHC [17] or residues 1120–1197 in soybean GmCHC [12], aligns to the residues 1112–1189 in ZmCHC1 or residues 1157–1234 in ZmCHC2 (Figure 2). Two sequence domains of great structural importance in CHC proteins are the trimerization domain and the light chain binding domain. Kirchhausen *et al* [17] initially suggested that the proline-rich region of the carboxy terminus (residues 1631–1675 in rat CHC) could be responsible for this interaction. Multiple sequence alignments showed that the trimerization domain and the light chain binding domain were well conserved in both maize CHCs (Figure 2). ZmCHC1 and ZmCHC2 proteins were phylogenetically classified into the monocots group in the plants CHCs cluster (Figure 3). Analysis of 3D models showed that the 3D structure of ZmCHC1 was more similar to those of Arabidopsis AtCHC1 and AtCHC2 (Figure 4), which were recently genetically and functionally characterized [11], whereas, the 3D structure of ZmCHC2 was strikingly similar to those of rice OsCHC1 and OsCHC2 (Figure 4), which were only deposited in the GenBank database. The high similarities of gene structure, protein sequences and 3D models among *ZmCHC1*, and Arabidopsis *AtCHC1* and *AtCHC2* suggest that they may have similar functions.

The constant expression of *ZmCHC1* and *ZmCHC2* in all organs examined (Figure 5) indicated that CHCs might play an important role in maize, but these proteins potentially have distinct functions with respect to development and defense against adverse conditions. Although clathrin is ubiquitous and plays a critical role in both endocytosis and exocytosis [20], *ZmCHC1* is predominantly expressed in maize roots (Figure 5).

In plants, endocytosis can be observed in many processes important for plant development, such as auxin transport [33], cytokinesis [34], root hair morphogenesis [35], pollen tube growth [36], self-incompatiblity responses [37], responses to pathogens [38], ABA responses [39], and responses to high boron levels [40,41]. During responses to pathogens, ABA, and high boron levels, the abundance of specific proteins in the plasma membrane is down-regulated through induction of their endocytosis [38–41]. The significance of endocytosis for these regulations is beyond doubt; however, functional data remain scarce. ABA, which controls ion transport and transpiration in plants under water stress [39], was reported to trigger the selective endocytosis of the KAT1, K^+^ channel protein in epidermal and guard cells in tobacco [39]. Boron (B) is essential for plants but toxic when present in excess [40]. Takano *et al.* [40] showed that endocytosis and degradation of BOR1, a plasma membrane transporter for B in plants were regulated by B availability, to avoid accumulation of toxic levels of B in shoots under high-B supply, while protecting the shoot from B deficiency under B limitation. To determine if the expressions of *ZmCHC1* and *ZmCHC2* genes during maize seedling growth were regulated by ABA or high boron supply, we analyzed the expression pattern of the two genes by real-time quantitative RT-PCR (Figure 6). The results showed a significant up-regulated expression of *ZmCHC1* instead of *ZmCHC2* in maize roots by ABA or high boron supply. The expression pattern of *ZmCHC1* in response to ABA might be due to the four predicted ABREs in its promoter sequence (Table 1).

Onelli *et al.* [42] showed that high concentrations of sodium azide inhibited the endocytic process, as observed an increase in particles on the plasma membrane and a decrease in large vesicles in tobacco protoplasts revealed by labelling with charged nanogold. Azides are known to affect ATP production [43]. The highest concentration of sodium azide which maintained cell viability was found to be 500 μmol/L; at 1 mmol/L sodium azide, tobacco protoplasts died [42]. In the present study, the expressions of *ZmCHC1* and *ZmCHC2* genes in maize plants were significantly down-regulated by 100 μmol/L sodium azide (Figure 6). The results of the sodium azide experiments further revealed the energy requirement of clathrin-mediated endocytosis [42]. Baluška *et al.* [44] showed that low temperature prevented intracellular internalization of cell wall pectins in all root meristem cells of maize. Onelli *et al.* [42] also suggested that low temperature slowed the endocytic process but did not stop it. In this study, a low temperature of 4 °C significantly down-regulated the expressions of *ZmCHC1* and *ZmCHC2* genes in maize plants (Figure 6), further suggesting that clathrin-mediated endocytosis in plants is sensitive to low temperatures [42].

When plants are attacked by pathogens, they defend themselves with an arsenal of defense mechanisms that includes *de novo* protein synthesis, which is regulated through a complex and interconnected network of signaling pathways. These pathways mainly involve the two signaling molecules SA and methyl jasmonate [45,46]. The SA pathway is associated with defense responses against pathogens. Endocytosis has been conceptualized in plant-microbe interactions as a requirement for defense signaling [47]. Ligand-induced receptor endocytosis appears as a component for activation of plant defense reactions [38]. In a previous study, Leborgne-Castel *et al.* [48] showed that the plant defense elicitor cryptogein, a protein secreted by the oomycete *Phytophthora cryptogea*, stimulated clathrin-mediated endocytosis (CME). In the present study, consistent with the typical predicted SA-responsive element (TCA-element) in the promoter sequence of *ZmCHC1* (Table 1), the expression of *ZmCHC1* instead of *ZmCHC2* in maize roots was significantly increased in response to SA (Figure 7), suggesting that *ZmCHC1* gene may be involved in the SA signaling pathway in maize defense responses. Tyrphostin A23 has been used to inhibit endocytosis: inhibition of clathrin-mediated endocytosis due to the interaction between tyrphostin A23 and the subunit μ2 of the AP-2 complex, one component of the membrane coat associated with clathrin, has been reported [49]. Tyrphostin A23 was utilized at a concentration of 30 μmol/L to inhibit internalization of the PIN auxin efflux carrier and different plasma membrane proteins in Arabidopsis roots [9,10] or 350 μmol/L to inhibit internalization of the transferrin in Arabidopsis protoplasts [10]. In the present study, the use of 30 μmol/L Tyrphostin A23 for 30 min did not affect the expressions of both *ZmCHC1* and *ZmCHC2* genes in maize roots (Figure 7).

In the present study, we first identify two maize genes encoding clathrin heavy chain in monocots via phylogenetic and structural analysis of amino acid sequences, and represent that their mRNA expressions are regulated by abiotic stimulus. These results are helpful to understand the roles of CME in plant adaptation to external environment. Although the importance of CME is unchallenged, its role in various physiological responses remains largely unclear. The precise role of CME in signal transduction has yet to be fully understood. For example, how does receptor endocytosis lead to the activation of signaling pathways, and how does CME induce this? Perhaps, the further understanding of the pathway will be facilitated by the advent of better inhibitors and a more complete understanding of its mechanistic details.

## 4. Experimental Section

### 4.1. Maize Plants and Growth Conditions

Seeds of maize (*Zea mays* L. “Zhongnuo No. 1”) were sown in pots with a potting mixture of vermiculite, perlite and soil (1:1:1), and grown in a growth chamber with 12 h of light from fluorescent and incandescent lamps (200 μmol m^−2^ s^−1^) followed by 12 h of darkness at 26 °C. Plants were watered daily with half-strength Murashige and Skoog nutrient solution [50].

### 4.2. Salicylic Acid, Abscisic Acid, High Boron, Sodium Azide, Tyrphostin A23, or Low Temperature Treatment of Maize Plants

For salicylic acid (SA), abscisic acid (ABA), high boron, sodium azide, or Tyrphostin A23 treatment, 12-day-old maize seedlings were removed from the soil mixture carefully to avoid injury, and then hydroponically grown in a solution containing 100 μmol/L SA for 24 h, 20 μmol/L ABA for 60 min, 100 μmol/L boric acid for 60 min, 100 μmol/L sodium azide for 2 min, 30 μmol/L Tyrphostin A23 (a commonly used inhibitor of CME [5,10]) for 30 min, or deionized water as a control, respectively. The low-temperature stress treatment was achieved by transferring the plants in pots to an incubator at 4 °C for 30 min. For all these treatments, plant samples were frozen in liquid nitrogen immediately following the treatments and stored at −80 °C until analysis. The entire experiments were biologically repeated at least three times.

### 4.3. Molecular Cloning of CHC Homolog from Maize

Total RNA was isolated from the frozen samples using the modified phenol-chloroform method as previously described [51]. For amplification of specific homologs encoding clathrin heavy chain (CHC) from maize, two degenerate forward primers (DP-F1, 5′-GTT AAG GCN TTY ATG ACI GC-3′; DP-F2, 5′-CAT TTC AAR TAY ATI GAR GCI GC-3′) were designed based on the conserved regions of the reported CHCs in database. The first-strand cDNA was synthesized from 2 μg of total RNA using SuperScript™ III RNase H^−^ Reverse Transcriptase (Invitrogen, Guangzhou, China) and an oligodT_30_ primer according to the manufacturer’s protocol. The cDNA was then used as template for PCR amplification using the primer sets of DP-F1 and oligodT_30_ or DP-F2 and oligodT_30_. The resulting PCR fragments were sequenced and compared with the reported *CHC* sequences in database. The missing 5′ ends of the amplified fragments were obtained by rapid amplification of cDNA ends (RACE) using the GeneRacer kit according to the manufacturer’s instructions (Invitrogen). The gene specific primers for *ZmCHC1* were 5GSP-R1-1 (5′-CTC AGC ATC GTA GAA GTT TGA CTC TCT GG-3′) (outer) and 5GSP-R1-2 (5′-CTT CTT TGA TCT GTC CAG TCC TCG C-3′) (inner); the gene-specific primers for *ZmCHC2* were 5GSP-R2-1 (5′-AAT CCA TGA CTC TGG ATG GAT CTG CC-3′) (outer) and 5GSP-R2-2 (5′-GAA GCT CAA TCA GTT CAT GTG GCA GG-3′) (inner). The promoter region sequence upstream of the start codon of *ZmCHC1* was cloned by using the Genome Walking Kit (TaKaRa) with three designed specific primers SP1 (5′-TCG GCT AGT ATG GAG CGG AAG GGC-3′), SP2 (5′-GAT CTG ATC TGA GGA GAG GGC CAG GG-3′) and SP3 (5′-GCC ACG TCG GTG CGG AGA AGG GG-3′).

To detect the possible transcript variants of *ZmCHC1* and *ZmCHC2* genes, RT-PCR was performed to amplify the sequence containing complete ORF with maize root cDNA as the template. The primer sets CHC1F-outer (5′-TTC TCC GCA CCG ACG TGG CGA TGT GTT-3′), CHC1R-outer (5′-TAC GGG TAA GAC TGA TTT CAC TCA CTC GCC-3′), and CHC1F-inner (5′-ACC GAC GTG GCG ATG TGT TGT GTC C-3′), CHC1R-inner (5′-CAA CCA TCT ACA ACT CCA ATG AAG TTC CAC-3′) were used for *ZmCHC1* gene RT-PCR performance; the primer sets CHC2F-outer (5′-CTC AAT GGA CAC CAT CTT TCC CAG A-3′), CHC2R-outer (5′-CTA CAA CTC CAA TGA AGT TTC CAC CTT CGC-3′), and CHC2F-inner (5′-GCT CTG TTG TGC TGT TTG GTT CCG TCG-3′), CHC2R-inner (5′-GGA CGA TTC AAA TCA TCG TCT AAG CTC-3′) were used for *ZmCHC2* gene RT-PCR performance. The primer sets CHC1F-outer, CHC1R-outer and CHC1F-inner, CHC1R-inner were also used to amplify the genomic DNA sequence corresponding to the coding sequence of *ZmCHC1* using a LA Taq DNA polymerase (TaKaRa, Dalian, China). The corresponding genomic DNA sequence of *ZmCHC2* was achieved by comparison of *ZmCHC2* cDNA sequence with the submitted maize chromosome sequence (GenBank accession no. CM000778). All the RT-PCR and genomic DNA-based PCR products were subcloned and sequenced from both strands to detect the possible transcript variants of maize *CHC* genes.

In all cloning experiments, PCR fragments were gel-purified with an Agarose Gel DNA Purification Kit (TaKaRa, Dalian, China) and were ligated into the pMD 19-T Vector (TaKaRa, Dalian, China). Plasmids harboring target fragments were isolated and were sequenced from both strands.

### 4.4. Sequence Analyses and Alignments

The routine sequence analysis was performed by using Gene Runner (Hastings Software, Inc., New York, NY, USA). Computer analysis of the DNA and amino acid sequences was carried out using the Basic Local Alignment Search Tool (BLAST) program at the National Center for Biotechnology Information Services [52]. Multiple alignments of the amino acid sequences of CHCs were performed using the Clustal W program in BioEdit software (Isis Pharmaceuticals, Inc., Carlsbad, CA, USA). The full-length CHC protein sequences were phylogenetically analyzed by using MEGA 4 software [25] with a bootstrapping set of 1000 replicates. 3D comparative protein structure models of CHCs were generated with the automatic modeling mode of SWISS-MODEL implemented on the SWISS-MODEL Workspace website (http://swissmodel.expasy.org/) [53,54]. The protein structures were color-coded. The prediction of the subcellular localization of some plant CHC proteins was performed by using the iPSORT algorithm [26] at the website: http://ipsort.hgc.jp/.

### 4.5. Real-Time Quantitative RT-PCR Performance

The isolated RNA by using the above mentioned method was treated with RNase-free DNase I (TaKaRa, Dalian, China) at 37 °C for 1 h to eliminate DNA contamination in real-time quantitative RT-PCR analysis. Two micrograms of total RNA and 200 ng of a random primer were used in reverse transcription (RT) through a cDNA synthesis kit (Invitrogen, Guangzhou, China) according to the manufacturer’s protocol. To investigate the expressions of *ZmCHC1* and *ZmCHC2* genes in maize, the gene-specific primers, GSP-1F (5′-GTG TCT ATG AAA CGT GAA ACA TGG GTC CG-3′) and GSP-1R (5′-TCT CTT TCG GCT AGT ATG GAG CGG AAG GGC-3′) for *ZmCHC1*, GSP-2F (5′-GAA TCG TCC CTG TGT AAG CGA AGG TGG-3′) and GSP-2R (5′-CAG CTC GCT CAA CCA ACC TAA TCA CCC-3′) for *ZmCHC2*, were designed to amplify a 229 base pairs (bp), or a 189 bp fragment of *ZmCHC1* or *ZmCHC2* cDNA for real-time quantitative PCR. As an internal control for normalization of target gene expression, the primers 18S-F (5′-TTC GAT GGT AGG ATA GGG GCC-3′) and 18S-R (5′-CTA TTG GAG CTG GAA TTA CCG CGG CTG C-3′) specific to the maize *18S rRNA* gene (GenBank accession no. AF168884) were used to amplify a fragment of 277 bp. Real-time quantitative PCRs were performed in the presence of Power SYBR green PCR Master Mix (Applied Biosystems, Guangzhou, China). Amplification was monitored in real-time with the MiniOpticon™ Real-Time PCR System (Bio-Rad, Shanghai, China). The products of real-time quantitative PCR were confirmed by determining the melt curves for the products at the end of each run, by analysis of the products using gel electrophoresis, and by sequencing. Quantification of the normalized gene expression was performed with the comparative cycle threshold (Ct) method [55]. Three biological and three technical replicates were performed for each experiment. The number of maize seedlings/organs of individual replicates was 11. All RT-PCR data were expressed as the mean ± standard error. Statistical differences of expressions of *ZmCHC1* and *ZmCHC2* among maize organs or various treatments were assessed by one-way analysis of variance (ANOVA) followed by the least significant difference (LSD) and Student-Neumann-Keuls (SNK) *post hoc* comparison. The analyses were performed with SPSS 13.0 software (SPSS Inc., Chicago, IL, USA). The threshold of significance was defined as *p* < 0.05.

## 5. Conclusions

Membrane vesicle traffic to and from the plasma membrane is essential for cellular homeostasis in all eukaryotes [56,57]. In plants, constitutive traffic to and from the plasma membrane has been implicated in maintaining the population of integral plasma membrane proteins and its adjustment to a variety of hormonal and environmental stimuli [57]. In the present study, the genes *ZmCHC1* and *ZmCHC2* encoding clathrin heavy chain in maize were cloned and characterized for the first time in monocot plants. The analysis of gene structure organization shows that *ZmCHC1* encodes a 1693-amino acid protein including 29 exons and 28 introns, and that *ZmCHC2* encodes a 1746-amino acid protein including 28 exons and 27 introns. The high similarities of gene structure, protein sequences and 3D models among *ZmCHC1*, and Arabidopsis *AtCHC1* and *AtCHC2* suggest that they may have similar functions. *ZmCHC1* gene is predominantly expressed in maize roots instead of ubiquitous expression of *ZmCHC2*. Consistent with the typical predicted SA-responsive element (TCA-element) and four predicted ABREs in the promoter sequence of *ZmCHC1*, the expression of *ZmCHC1* instead of *ZmCHC2* in maize roots is significantly up-regulated by SA or ABA, suggesting that *ZmCHC1* gene may be involved in the SA signaling pathway in maize defense responses. The significant down-regulation of the expressions of *ZmCHC1* and *ZmCHC2* genes by azide or cold treatment further reveals the energy requirement of clathrin-mediated endocytosis, and suggests that clathrin-mediated endocytosis in plants is sensitive to low temperatures.

## Figures and Tables

**Table 1:** Main *cis*-acting elements in promoter region sequences of *ZmCHC1* and *ZmCHC2* genes predicted in PlantCARE, a database of plant *cis*-acting regulatory elements.

*cis*-Acting elements	Position (upstream of the start codon of *ZmCHC1* or *ZmCHC2*)		Core sequence		Function
	
	*ZmCHC1*	*ZmCHC2*	*ZmCHC1*	*ZmCHC2*	
TCA-element	−403 to −412		GAGAAGAAGA		Salicylic acid responsiveness

ABRE	−152 to −143	−880 to −875	CCGACGTGGC	CACGTG	Abscisic acid responsiveness
	−1163 to −1158		CGTGGA		
	−1189 to −1180		TGGGGGTGGC		
	−1441 to −1436		TACGTG		

Box-W1	−1470 to −1475	−1279 to −1274	TTGACC	TTGACC	Fungal elicitor responsiveness

TGACG-motif	−66 to −70 −847 to −843	−945 to −949	TGACG TGACG	TGACG	Methyl jasmonate responsiveness
		−1065 to −1069		TGACG	
		−1245 to −1241		TGACG	

## References

[1] Conner S.D., Schmid S.L. (2003). Regulated portals of entry into the cell. Nature.

[2] Murphy A.S., Bandyopadhyay A., Holstein S.E., Peer W.A. (2005). Endocytotic cycling of PM proteins. Annu. Rev. Plant Biol.

[3] Wieffer M., Maritzen T., Haucke V. (2009). SnapShot: Endocytic trafficking. Cell.

[4] McMahon H.T., Boucrot E. (2011). Molecular mechanism and physiological functions of clathrin-mediated endocytosis. Nat. Rev. Mol. Cell Biol.

[5] Chen X., Irani N.G., Friml J. (2011). Clathrin-mediated endocytosis: The gateway into plant cells. Curr. Opin. Plant Biol.

[6] Adam T., Bouhidel K., Der C., Robert F., Najid A., Simon-Plas F., Leborgne-Castel N. (2012). Constitutive expression of clathrin hub hinders elicitor-induced clathrin-mediated endocytosis and defense gene expression in plant cells. FEBS Lett.

[7] Pearse B.M. (1976). Clathrin: A unique protein associated with intracellular transfer of membrane by coated vesicles. Proc. Natl. Acad. Sci. USA.

[8] Kirchhausen T. (2009). Imaging endocytic clathrin structures in living cells. Trends Cell Biol.

[9] Dhonukshe P., Aniento F., Hwang I., Robinson D.G., Mravec J., Stierhof Y.D., Friml J. (2007). Clathrin-mediated constitutive endocytosis of PIN auxin efflux carriers in Arabidopsis. Curr. Biol.

[10] Robert S., Kleine-Vehn J., Barbez E., Sauer M., Paciorek T., Baster P., Vanneste S., Zhang J., Simon S., Covanova M. (2010). ABP1 mediates auxin inhibition of clathrin-dependent endocytosis in Arabidopsis. Cell.

[11] Kitakura S., Vanneste S., Robert S., Lofke C., Teichmann T., Tanaka H., Friml J. (2011). Clathrin mediates endocytosis and polar distribution of PIN auxin transporters in Arabidopsis. Plant Cell.

[12] Blackbourn H.D., Jackson A.P. (1996). Plant clathrin heavy chain: Sequence analysis and restricted localisation in growing pollen tubes. J. Cell Sci.

[13] Fujimoto M., Arimura S., Ueda T., Takanashi H., Hayashi Y., Nakano A., Tsutsumi N. (2010). Arabidopsis dynamin-related proteins DRP2B and DRP1A participate together in clathrin-coated vesicle formation during endocytosis. Proc. Natl. Acad. Sci. USA.

[14] Food and Agriculture Organization of the United Nations http://faostat.fao.org.

[15] Tester M., Langridge P. (2010). Breeding technologies to increase crop production in a changing world. Science.

[16] Lescot M., Ddhais P., Thijs G., Marchal K., Moreau Y., van de Peer Y., Rouzd P., Rombauts S. (2002). PlantCARE: A database of plant *cis*-acting regulatory elements and a portal to tools for *in silico* analysis of promoter sequences. Nucleic Acids Res.

[17] Kirchhausen T., Harrison S.C., Chow E.P., Mattaliano R.J., Ramachandran K.L., Smart J., Brosius J. (1987). Clathrin heavy chain: Molecular cloning and complete primary structure. Proc. Natl. Acad. Sci. USA.

[18] Lemmon S.K., Pellicena-Palle A., Conley K., Freund C.L. (1991). Sequence of the clathrin heavy chain from *Saccharomyces cerevisiae* and requirement of the COOH terminus for clathrin function. J. Cell Biol.

[19] Doge G.R., Kovalszky I., McBride O.W., Yi H.F., Chu M., Saitta B., Stokes D., Iozzo R.V. (1991). Human clathrin heavy chain (CLTL): Partial molecular cloning, expression, and mapping of the gene to human chromosome 17q11-qter. Genomics.

[20] Sirotkin H., Morrow B., DasGupta R., Goldberg R., Patanjali S.R., Shi G., Cannizzaro L., Shprintzen R., Weissman S.M., Kucherlapati R. (1996). Isolation of a new clathrin heavy chain gene with muscle-specific expression from the region commonly deleted in velo-cardio-facial syndrome. Hum. Mol. Genet..

[21] Bazinet C., Katzen A.L., Morgan M., Mahowald A.P., Lemmon S.K. (1993). The Drosophila clathrin heavy chain gene: Clathrin function is essential in a multicellular organism. Genetics.

[22] Wingen C., Stumpges B., Hoch M., Behr M. (2009). Expression and localization of clathrin heavy chain in *Drosophila melanogaster*. Gene Expr. Patterns.

[23] Hölzenspies J.J., Roelen B.A.J., Colenbrander B., Romijn R.A.P., Hemrika W., Stoorvogel W., van Haeften T. (2010). Clathrin is essential for meiotic spindle function in oocytes. Reproduction.

[24] Nathke I.S., Heuser J., Lupas A., Stockton J., Turck C.W., Brodsky F.M. (1992). Folding and trimerisation of clathrin subunits at the triskelion hub. Cell.

[25] Tamura K., Dudley J., Nei M., Kumar S. (2007). MEGA4: Molecular evolutionary genetics analysis (MEGA) software version 4.0. Mol. Biol. Evol.

[26] Bannai H., Tamada Y., Maruyama O., Nakai K., Miyano S. (2002). Extensive feature detection of *N*-terminal protein sorting signals. Bioinformatics.

[27] Bari R., Jones J.D. (2009). Role of plant hormones in plant defence responses. Plant Mol. Biol.

[28] An C., Mou Z. (2011). Salicylic acid and its function in plant immunity. J. Integr. Plant Biol.

[29] Vernooij B., Friedrich L., Morse A., Rest R., Kolditz-Jawahar R., Ward E., Uknef S., Kessmann H., Ryalf J. (1994). Salicylic acid is not the translocated signal responsible for inducing systemic acquired resistance but is required for signal transduction. Plant Cell.

[30] Pérez-Gómez J., Moore I. (2007). Plant endocytosis: It is clathrin after all. Curr. Biol..

[31] Geldner N., Jurgens G. (2006). Endocytosis in signalling and development. Curr. Opin. Plant Biol.

[32] Holstein S.E. (2002). Clathrin and plant endocytosis. Traffic.

[33] Dhonukshe P., Tanaka H., Goh T., Ebine K., Mähönen A.P., Prasad K., Blilou I., Geldner N., Xu J., Uemura T. (2008). Generation of cell polarity in plants links endocytosis, auxin distribution and cell fate decisions. Nature.

[34] Boutté Y., Frescatada-Rosa M., Men S., Chow C.M., Ebine K., Gustavsson A., Johansson L., Ueda T., Moore I., Jurgens G. (2010). Endocytosis restricts Arabidopsis KNOLLE syntaxin to the cell division plane during late cytokinesis. EMBO J.

[35] Takeda S., Gapper C., Kaya H., Bell E., Kuchitsu K., Dolan L. (2008). Local positive feedback regulation determines cell shape in root hair cells. Science.

[36] Zhao Y., Yan A., Feijó J.A., Furutani M., Takenawa T., Hwang I., Fu Y., Yang Z. (2010). Phosphoinositides regulate clathrindependent endocytosis at the tip of pollen tubes in Arabidopsis and tobacco. Plant Cell.

[37] Ivanov R., Gaude T. (2009). Endocytosis and endosomal regulation of the S-receptor kinase during the self-incompatibility response in *Brassica oleracea*. Plant Cell.

[38] Robatzek S., Chinchilla D., Boller T. (2006). Ligand-induced endocytosis of the pattern recognition receptor FLS2 in Arabidopsis. Genes Dev.

[39] Sutter J.U., Sieben C., Hartel A., Eisenach C., Thiel G., Blatt M.R. (2007). Abscisic acid triggers the endocytosis of the Arabidopsis KAT1 K^+^ channel and its recycling to the plasma membrane. Curr. Biol.

[40] Takano J., Miwa K., Yuan L., von Wirén N., Fujiwara T. (2005). Endocytosis and degradation of BOR1, a boron transporter of Arabidopsis thaliana, regulated by boron availability. Proc. Natl. Acad. Sci. USA.

[41] Takano J., Tanaka M., Toyoda A., Miwa K., Kasai K., Fuji K., Onouchi H., Naito S., Fujiwara T. (2010). Polar localization and degradation of Arabidopsis boron transporters through distinct trafficking pathways. Proc. Natl. Acad. Sci. USA.

[42] Onelli E., Prescianotto-Baschong C., Caccianiga M., Moscatelli A. (2008). Clathrin-dependent and independent endocytic pathways in tobacco protoplasts revealed by labeling with charged nanogold. J. Exp. Bot.

[43] Prescianotto-Baschong C., Riezman H. (1998). Morphology of the yeast endocytic pathway. Mol. Biol. Cell.

[44] Baluska F., Hlavacka A., Samaj J., Palme K., Robinson D.G., Matoh T., McCurdy D., Menzel D., Volkmann D. (2002). F-actin dependent endocytosis of cell wall pectins in meristematic root cells. Insights from Brefeldin A-induced compartments. Plant Physiol.

[45] Koornnef A., Pieterse C.M. (2008). Cross talk in defence signaling. Plant Physiol.

[46] Mika A., Boenisch M.J., Hopff D., Luthje S. (2010). Membrane-bound guaiacol peroxidases from maize (*Zea mays* L.) roots are regulated by methyl jasmonate, salicylic acid, and pathogen elicitors. J. Exp. Bot..

[47] Leborgne-Castel N., Adam T., Bouhidel K. (2010). Endocytosis in plant-microbe interactions. Protoplasma.

[48] Leborgne-Castel N., Lherminier J., Der C., Fromentin J., Houot V., Simon-Plas F. (2008). The plant defense elicitor cryptogein stimulates clathrin-mediated endocytosis correlated with reactive oxygen species production in Bright Yellow-2 tobacco cells. Plant Physiol.

[49] Banbury D.N., Oakley J.D., Sessions R.B., Banting G. (2003). Tyrphostin A23 inhibits internalization of the transferrin receptor by perturbing the interaction between tyrosine motifs and the medium chain subunit of the AP-2 adaptor complex. J. Biol. Chem.

[50] Murashige T., Skoog F. (1962). A revised medium for rapid growth and bioassay with tobacco tissue cultures. Physiol. Plant.

[51] Wan X.R., Li L. (2005). Molecular cloning and characterization of a dehydration-inducible cDNA encoding a putative 9-*cis*-epoxycarotenoid dioxygenase in *Arachis hypogaea* L. DNA Seq.

[52] Altschul S.F., Gish W., Miller W., Myers E.W., Lipman D.J. (1990). Basic local alignment search tool. J. Mol. Biol.

[53] Schwede T., Kopp J., Guex N., Peitsch M.C. (2003). SWISS-MODEL: An automated protein homology-modeling server. Nucleic Acids Res.

[54] Arnold K., Bordoli L., Kopp J., Schwede T. (2006). The SWISS-MODEL workspace: A web-based environment for protein structure homology modeling. Bioinformatics.

[55] Muller P.Y., Janovjak H., Miserez A.R., Dobbie Z. (2002). Processing of gene expression data generated by quantitative real-time RT-PCR. Biotechniques.

[56] Jahn R., Lang T., Sudhof T.C. (2003). Membrane fusion. Cell.

[57] Surpin M., Raikhel N. (2004). Traffic jams affect plant development and signal transduction. Nat. Rev. Mol. Cell Biol.

